# Thrombocytosis and Thrombocytopenia in Treatment-Naïve Rheumatoid Arthritis Patients: A Single-Centre Retrospective Data-Based Study From Pakistan

**DOI:** 10.7759/cureus.73112

**Published:** 2024-11-06

**Authors:** Nauman Ismat Butt, Muhammad Sohail Ajmal Ghoauri, Barak Waris, Muhammad Umair Javed, Dure Sabeh, Fahad Qaisar

**Affiliations:** 1 Internal Medicine/Rheumatology, Azra Naheed Medical College, Superior University, Lahore, PAK; 2 Neurology, Bahawal Victoria Hospital, Quaid-e-Azam Medical College, Bahawalpur, PAK; 3 Medicine and Allied Health Sciences, Chaudhry Muhammad Akram Teaching and Research Hospital, Lahore, PAK; 4 Medicine and Allied Health Sciences, Azra Naheed Medical College, Superior University, Lahore, PAK; 5 Medicine, Bahawal Victoria Hospital, Quaid-e-Azam Medical College, Bahawalpur, PAK

**Keywords:** anti-ccp antibodies, disease activity score (das-28), ra factor, rheumatoid arthriitis, thrombocytopenia, thrombocytosis

## Abstract

Background and objective

This study aims to investigate thrombocytosis and thrombocytopenia in treatment-naïve rheumatoid arthritis (RA) patients, addressing the lack of data on their prevalence and clinical significance. By focusing on untreated patients, this study seeks to clarify the disease's natural course without confounding factors from prior therapies. The research is particularly important in the Pakistani context, where regional variations in RA presentation are underexplored. Findings are expected to enhance understanding of these hematological changes, ultimately guiding improved clinical management strategies for RA.

Methods

The present retrospective data-based study was conducted at the Department of Medicine & Allied Health Sciences, Azra Naheed Medical College, Superior University, Lahore, Pakistan. The 2010 American College of Rheumatology (ACR) diagnostic criteria were used to define RA. Thrombocytosis was defined as a platelet count greater than 400×10^9^/L. Thrombocytopenia was defined as a platelet count less than 150×10^9^/L. The Disease Activity Score in 28 joints (DAS-28 score) was used to categorize the disease severity of RA. Retrospective data were collected from the medical records of 165 patients with RA. Records from January 2023 to December 2023 were included. The assessment included demographic information, rheumatoid factor status, anti-CCP antibody status, disease severity measured by the DAS-28 score, and complete blood count (CBC) results, such as hemoglobin, white blood cell (WBC) count, and platelet count. Data entry and analysis were conducted using IBM SPSS Statistics software, version 23 (IBM Corp., Armonk, NY).

Results

The mean age of the patients was 42.5±13.6 years, with 25 (15.2%) male and 140 (84.8%) female patients. The RA factor (RAF) was positive in 128 (77.6%) and the anti-CCP antibody in 102 (61.8%). The mean DAS-28 score was 4.5±1.4, with 113 (68.5%) patients having mild-to-moderate disease and 52 (31.5%) patients having severe disease. Mean hemoglobin, WBC, and platelet counts were 12.0±1.4 g/dl, 9.0±2.7 ×10^9^/L, and 352.6±110.4 ×10^9^/L, respectively. Thrombocytosis was seen in 52 (31.5%) patients, while thrombocytopenia was seen in 11 (6.7%).

Conclusion

Thrombocytosis was seen in almost one-third of RA patients, having a significant association with age, the RAF factor positivity, and anti-CCP antibody negativity but not with gender and disease severity. Thrombocytopenia was relatively uncommon.

## Introduction

Platelets are produced by megakaryocytes, while their formation and maturation is controlled by thrombopoietin in the bone marrow. The primary role of platelets is blood clot formation and thrombosis, but they are also involved in the immune system, inflammation, wound healing, and cancer biology [1]. The typical platelet count is 150,000 to 450,000/mm^3 ^(or 150×10^9^/L to 400×10^9^/L). In thrombocytopenia, the platelet count is less than 150,000/mm^3^ (or 150×10^9^/L), while in thrombocytosis, the platelet count is greater than 400,000/mm^3^ (or 400×10^9^/L). Among other factors, platelets are a component of the extrinsic clotting pathway. When undamaged, the vascular endothelium is usually smooth, secreting nitric oxide, which prevents platelet adherence to the endothelium [2]. When the vascular endothelial lining is damaged, platelets adhere to it and secrete adhesive proteins, nucleotides, growth factors, and various pro-coagulants that cause thrombosis and blood clot formation [3]. Furthermore, the fibrin mesh generated as a consequence of the intrinsic clotting pathway adds strength to this platelet plug, preventing further damage [2,3].

The main clinical consequence of thrombocytopenia is bleeding due to poor platelet plug formation and inadequate primary hemostasis. Thrombocytopenia may be caused by decreased platelet production in the bone marrow, increased platelet destruction, and sequestration, including causes such as primary idiopathic immune thrombocytopenia (ITP), drugs, infections, autoimmune rheumatic disorders, and hematologic malignancies [4,5]. On the other hand, thrombocytosis can lead to excessive platelet plug formation and active intravascular coagulation, causing thromboembolism [6].

Rheumatoid arthritis (RA) has a prevalence of 1%-3% in the general population, is usually more common in females, and has a genetic predisposition [7]. A chronic autoimmune disease, RA affects the joints primarily, but it can lead to systemic manifestations, including hematological cytopenia and cytosis [6,7]. Tripathi et al. reported thrombocytosis to be present in 18.9% of patients with RA [8]. Hutchinson et al. reported thrombocytosis to be seen in as many as 52% of patients with RA [6]. It was further reported that an increased platelet count was associated with anemia, severe disease with extra-articular manifestations, and RA factor (RAF) positivity [6]. Various mechanisms that thrombocytosis in RA include acute and chronic blood loss due to chronic anemia of active disease, resulting in increased erythropoietin and thrombopoietin levels that are chemically similar [6]. Another explanation is compensatory increased platelet production as a result of increased platelet consumption in RA. It has been postulated that thrombocytosis can be a consequence of immunological responses secondary to micro-thromboses in the chronically inflamed synovium in RA [6].

Thrombocytopenia is uncommon in RA, having an incidence varying from 0.1% to 10% [1,9]. The most common cause of thrombocytopenia in RA patients on therapy is drug-induced thrombocytopenia [10]. The majority of conventional disease-modifying anti-rheumatic drugs (DMARDs) such as methotrexate, sulfasalazine, and leflunomide can lead to bone marrow suppression and thrombocytopenia, among other adverse effects [9,11]. Drug-dependent antibodies against platelet membrane glycoproteins (IIb/IIIa and Ib/IX) can also result in impaired production and reduced platelet lifespan, resulting in thrombocytopenia [12]. Rarely, ITP can also be seen in RA, which may be more resistant to treatment as compared to ITP without RA [13]. Vitamin B12 or folic acid, can also be a cause of thrombocytopenia, but most patients with RA take supplementation when using DMARD therapy.

The rationale for the present study is grounded in several critical factors that underscore the importance of investigating thrombocytosis and thrombocytopenia in treatment-naïve RA patients. Firstly, thrombocytopenia is a recognized complication in various autoimmune diseases, including RA, yet comprehensive data on its prevalence among treatment-naïve patients remains limited. Understanding the frequency and implications of thrombocytopenia can help clinicians identify at-risk patients and manage their care more effectively. Secondly, thrombocytosis is often observed in inflammatory conditions, but its specific prevalence and clinical significance in the context of RA, particularly among those who have not yet received treatment, have not been thoroughly studied. This lack of data can hinder appropriate diagnosis and management strategies. Furthermore, the paucity of research from Pakistan on these hematological conditions highlights a critical gap in the understanding of how RA presents in diverse populations. Regional studies are essential to inform local clinical practices and public health strategies, as disease manifestations and associated risks can vary significantly based on genetic, environmental, and lifestyle factors.

By focusing on treatment-naïve patients, this study aims to eliminate confounding variables related to prior therapies, providing a clearer picture of the disease’s natural course. This approach will enhance the understanding of the relationship between RA and hematological changes, ultimately guiding clinicians in making timely and informed decisions about patient care. Overall, the findings from this study are expected to contribute to a more nuanced understanding of thrombocytosis and thrombocytopenia in RA, thereby bridging an important knowledge gap and supporting the development of targeted management strategies in the Pakistani context and beyond.

## Materials and methods

The present retrospective data-based study was conducted at the Department of Medicine & Allied Health Sciences, Azra Naheed Medical College, Superior University, Lahore, Pakistan, to investigate the frequency of thrombocytosis and thrombocytopenia among treatment-naïve patients of rheumatoid arthritis. The 2010 American College of Rheumatology (ACR) Diagnostic Criteria were used to define RA (Appendix A) [14]. Normal platelet count is from 150×10^9^/L to 400×10^9^/L. Thrombocytosis was defined as a platelet count greater than 400×10^9^/L. Thrombocytopenia was defined as a platelet count less than 150×10^9^/L. The Disease Activity Score in 28 joints (DAS-28) score was used to categorize the disease severity of RA (Appendix B) [15]. Severe disease was defined as having a DAS-28 score greater than 5.1, while mild to moderate disease had a DAS-28 score from 2.7 to 5.1. Keeping a margin of error of 6% and a confidence interval of 95%, a sample size of 165 patients was required, keeping the expected frequency of thrombocytosis at 18.9% and the expected frequency of thrombocytopenia at 10% [1,8].

The present study was conducted in accordance with the ethical standards laid down in the 1964 Declaration of Helsinki, revised in the year 2000. The study was approved by the Institutional Review Board of Azra Naheed Medical College, Superior University, prior to data collection (IRB/ANMC/2024/03). To protect patient confidentiality, all data were anonymized before analysis, and access was restricted to authorized personnel only. These measures were implemented to uphold ethical standards and safeguard participants' rights throughout the research process. Retrospective data of the initial first visit of 165 treatment-naïve patients, aged 21 to 80 years of both genders with RA was assessed. Records from January 2023 to December 2023 were included. Medical records of patients with incomplete data, patients with a prior history of platelet disorders, and patients with histories of conventional or biologic DMARD use at the time of first presentation were excluded.

Basic demographic and clinical information, including age, gender, RA factor status, anti-CCP antibody status, parameters, and disease severity according to the DAS-28 score, and complete blood count (CBC) results, including hemoglobin, white blood cell (WBC) count, and platelet count, were assessed and recorded. In this study, data were collected from the medical records of patients diagnosed with RA at Chaudhary Muhammad Akram Teaching and Research Hospital, Azra Naheed Medical College in Lahore, Pakistan. Data extraction involved both electronic health records (EHRs) and manual methods. Electronic health records were used for efficient access to patient information while manual extraction was employed to ensure comprehensive capture of patient record details, ensuring comprehensive patient details were captured for analysis and reporting. The research team received training to maintain consistency and accuracy in data handling. Quality control measures included cross-verifying a subset of records to enhance data reliability. Missing data were addressed using a systematic approach to maintain the integrity of the dataset. Any incomplete records were evaluated to determine the extent and pattern of the missing information. Depending on the nature of the missing data, we employed multiple imputation techniques to estimate missing values while minimizing bias. For cases with critical missing information that could affect the study's outcomes, those records were excluded from the analysis. This approach ensured that the final dataset remained robust and reliable for statistical analysis.

IBM SPSS Statistics software, version 23 (IBM Corp., Armonk, NY) was employed for data entry and analysis. Mean and standard deviation were calculated for quantitative variables, while qualitative variables were presented as percentages and frequency. Confounders and effect modifiers were controlled via stratification, with p ≤ 0.05 significant. The chi-square test was applied, while Fisher's exact test was applied when the expected count was less than 5%, followed by logistic regression analysis. Binary logistic regression was chosen for this study due to the binary nature of the outcome variable, allowing for effective modeling of the relationship between predictor variables and the outcome. This method also enables control for potential confounding variables, enhancing the robustness of our findings. With an adequate sample size of 165 treatment-naïve RA patients, the analysis possesses sufficient statistical power to detect meaningful associations, making this approach both methodologically sound and clinically relevant.

## Results

Medical records of 165 treatment-naïve RA patients were assessed retrospectively, with a mean age of 42.5±13.6 years ranging from 21 to 76 years. Seventy-nine (47.9%) patients were aged 40 years or younger, while 86 (52.1%) patients were aged 41 years or older. Twenty-five (15.2%) patients were male, while 140 (84.8%) patients were female. The RAF was positive in 128 (77.6%), and the anti-CCP antibody was seen in 102 (61.8%). The mean erythrocyte sedimentation rate (ESR) was 37.1±23.8 mm/hr. The mean visual analog score (VAS) score, tender joint count, and swollen joint count were 4.6±2.7, 5.2±4.0, and 3.0±2.8, respectively. The mean DAS-28 score was 4.5±1.4, with 113 (68.5%) patients having mild-to-moderate disease (DAS-28 score 2.7-5.1), while 52 (31.5%) patients had severe disease (DAS-28 score >5.1). The mean hemoglobin, WBC, and platelet counts were 12.0±1.4 g/dl, 9.0±2.7 ×10^9^/L, and 352.6±110.4 ×10^9^/L, respectively. Thrombocytosis was seen in 52 (31.5%) patients, while thrombocytopenia was seen in 11 (6.7%).

Stratification of data with regards to thrombocytosis is shown in Table 1, revealing a significant association with increasing age, RAF factor positivity, and anti-CCP antibody negativity but not with gender or disease severity. Age is notably relevant, with 17 (21.5%) of individuals aged 40 or less exhibiting thrombocytosis compared to 35 (40.7%) in those aged 41 or more (p-value = 0.008). Additionally, the presence of RAF shows a strong correlation, as only 33 (25.8%) of RA-positive patients had thrombocytosis versus 19 (51.4%) of RA-negative patients (p-value = 0.003). Similarly, anti-CCP antibodies are significantly associated, with just 21 (20.6%) of positive cases showing thrombocytosis compared to 31 (49.2%) of negative cases (p-value < 0.001). Stratification of data with regards to thrombocytopenia is shown in Table 2, revealing no significant association with age, gender, RA factor positivity, anti-CCP antibody positivity, or disease severity.

**Table 1:** Stratification of clinical variables according to thrombocytosis *p-value <0.05, significant

Clinical variables		Thrombocytosis		p-value
		Present	Absent	
Gender	Female	44 (31.4%)	96 (68.6%)	0.955
	Male	08 (32.0%)	17 (68.0%)	
Age (years)	40 or less	17 (21.5%)	62 (78.5%)	0.008*
	41 or more	35 (40.7%)	51 (59.3%)	
Rheumatoid arthritis factor	Positive	33 (25.8%)	95 (74.2%)	0.003*
	Negative	19 (51.4%)	18 (48.6%)	
Anti-CCP antibodies	Positive	21 (20.6%)	81 (79.4%)	<0.001*
	Negative	31 (49.2%)	32 (50.8%)	
Disease activity	Mild-to-moderate	35 (31.0%)	78 (69.0%)	0.825
	Severe	17 (32.7%)	35 (67.3%)	

**Table 2:** Stratification of clinical variables according to thrombocytopenia

Clinical variables		Thrombocytopenia		p-value
		Present	Absent	
Gender	Female	09 (6.4%)	131 (93.6%)	0.772
	Male	02 (8.0%)	23 (92.0%)	
Age (years)	40 or less	08 (10.1%)	71 (89.9%)	0.120
	41 or more	03 (3.5%)	83 (96.5%)	
Rheumatoid arthritis factor	Positive	08 (6.2%)	120 (93.8%)	0.690
	Negative	03 (8.1%)	34 (91.9%)	
Anti-CCP antibodies	Positive	08 (7.8%)	94 (92.2%)	0.535
	Negative	03 (4.8%)	60 (95.2%)	
Disease activity	Mild-to-moderate	09 (8.0%)	104 (94.0%)	0.505
	Severe	02 (3.8%)	50 (96.2%)	

The logistic regression analysis reveals that among the variables examined, age, RAF, and anti-CCP antibodies significantly influence thrombocytosis, as indicated by their p-values (RAF: p = 0.044; CCP: p = 0.002; groups' age: p = 0.018). Specifically, for each unit increase in RA factor, the odds of thrombocytosis decrease by approximately 57% (Exp(B) = 0.429), while a unit increase in anti-CCP antibodies reduces the odds by about 68% (Exp(B) = 0.318). Additionally, an increase in age is associated with a 62% decrease in the odds of the outcome (Exp(B) = 0.381). In contrast, gender and disease severity of RA were not statistically significant predictors as depicted in Table 3.

**Table 3:** Logistic regression analysis assessing the impact of various clinical variables on thrombocytosis *p-value <0.05, significant; S.E: standard error

Variables	Beta coefficient (B)	S.E.	Wald	df	p-value	Exp(B)	95% C.I. for Exp(B)	
							Lower	Upper
Gender	0.76	0.531	0.21	1	0.886	1.079	0.381	3.054
Age	-0.966	0.410	5.558	1	0.018*	0.381	0.171	0.850
Rheumatoid arthritis factor	-0.846	0.421	4.043	1	0.044*	0.429	0.188	0.979
Anti-CCP antibodies	-1.146	0.375	9.319	1	0.002*	0.318	0.152	0.664
Disease activity	-0.268	0.425	0.397	1	0.529	0.765	0.332	1.761

The logistic regression analysis indicates that none of the variables, including gender, age, RAF, anti-CCP, or RA disease severity, were statistically significant at the conventional alpha level (p < 0.05). While age approached significance with a p-value of 0.059, suggesting a potential relationship, it did not reach the threshold for strong evidence. The odds ratio for age (Exp(B) = 3.967) implies that a unit increase in this variable could nearly quadruple the odds of the outcome, although the confidence interval is quite wide (Lower: 0.947, Upper: 16.612), reflecting uncertainty. Similarly, the odds ratios for other variables, gender (Exp(B) = 1.339), RAF (Exp(B) = 0.581), anti-CCP antibodies (Exp(B) = 1.509), and disease severity (Exp(B) = 3.293), suggest varying potential impacts on the outcome, but none are statistically significant as depicted in Table 4.

**Table 4:** Logistic regression analysis assessing the impact of various clinical variables on thrombocytopenia S.E: standard error

Variables	Beta coefficient (B)	S.E.	Wald	df	p-value	Exp(B)	95% C.I. for Exp(B)	
							Lower	Upper
Gender	0.292	0.873	0.112	1	0.738	1.339	0.242	7.410
Age	1.378	0.731	3.557	1	0.059	3.967	0.947	16.612
Rheumatoid arthritis factor	-0.544	0.765	0.505	1	0.477	0.581	0.130	2.602
Anti-CCP antibodies	0.411	-.768	0.287	1	0.592	1.509	0.335	6.800
Disease activity	1.192	0.833	2.046	1	0.153	3.293	0.643	16.861

## Discussion

Abnormal expression and dysfunction of proinflammatory cytokines in chronic autoimmune and inflammatory disorders such as RA can result in abnormalities of cell lines and even unregulated clonal proliferation [16]. This has led to speculations of a substantial link between thrombocytosis, thrombocytopenia, and autoimmune inflammatory disorders. Furthermore, rheumatic diseases such as RA and SLE have been associated with a higher risk of hematological malignancies such as lymphoproliferative disorders [17,18]. Thrombocytosis has been frequently reported with active joint disease in RA in relation to active joint disease. However, extremely high and persistently elevated levels of platelets in RA may be associated with myeloproliferative disorders, which require specific treatment in addition to usual RA therapy [19]. High levels of circulating platelets can result in spontaneous aggregation, increasing the propensity of arterial and venous thrombosis, gangrene, and other complications, including transient ischemic attack (TIA), stroke, and coronary artery disease [19]. It is therefore important to differentiate reactive thrombocytosis in RA patients from thrombocytosis caused by myeloproliferative disorders.

In the present study, thrombocytosis was seen in 52 (31.5%) treatment-naïve RA patients, which is higher when compared to previous studies suggesting more severe inflammatory responses in our patients, which may be due to more severe or active disease, potentially driven by underlying inflammatory processes. Tripathi et al. reported thrombocytosis in 18.9% of patients with RA [8]. In the study by Al-Ghamdi et al., thrombocytosis was seen in 16%, while thrombocytopenia (Felty’s syndrome) was in 1% [20]. Hutchinson et al. demonstrated thrombocytosis in 52% of RA patients, showing that an increased platelet count was associated with anemia, severe disease with extra-articular manifestations, and RA factor positivity [6]. In the present study, thrombocytosis had a significant association with increasing age, RAF positivity, and anti-CCP antibody negativity but not with gender or disease severity. The significant associations with increasing age and RAF positivity indicate that older patients and those with positive serological markers may experience heightened inflammation, leading to increased platelet production. The negative correlation with anti-CCP antibodies may point to a distinct disease phenotype where traditional markers do not align with platelet responses.

In the present study, thrombocytopenia was seen in 11 (6.7%) treatment-naïve RA patients, revealing no significant association with age, gender, RA factor positivity, anti-CCP antibody positivity, or disease severity. This highlights thrombocytopenia as a less common and potentially more random finding in RA. Previous studies show variable frequency of thrombocytopenia in RA patients from 0.1% to 10% [1,9]. The prevalence of thrombocytopenia in our study aligns with existing literature, indicating it is a less common complication in RA. The lack of significant associations with demographic or serological factors suggests that thrombocytopenia may arise from other, less predictable mechanisms rather than being directly linked to disease activity. Overall, our results emphasize the need for careful monitoring of platelet counts in RA, as thrombocytosis may signal increased disease activity while thrombocytopenia could require further evaluation in affected patients. These findings highlight the complexity of platelet dynamics in RA and the importance of individualized patient assessment to identify those at higher risk for complications related to altered platelet counts.

Thrombocytosis and thrombocytopenia should be included when determining extra-articular manifestations of RA, as these may have a significant impact on the disease [20]. Approximately 40% of RA patients face extra-articular manifestations, and their presence is linked to severe disease and higher mortality [21,22]. Patients with RA have a four-fold increase in mortality as compared to the general population [23]. Other than hemopoietic cells, these extra-articular manifestations may involve any organ system, including the lungs, kidneys, heart, and brain [24,25]. Furthermore, no reliable factors have been identified to predict the development of extra-articular manifestations in RA. Some factors thought to play a role include male sex, HLA-related epitope genes, RAF, anti-CCP antibodies, and environmental factors (cigarette smoking) [26,27].

The present study has several limitations that warrant careful consideration. Firstly, it was conducted at a single institution, which may affect the generalizability of the results to broader populations. Additionally, the relatively small sample size may limit the statistical power of our findings, potentially masking other relevant associations. Furthermore, the reliance on retrospective data introduces inherent biases and may affect the accuracy of the clinical variables assessed. Despite these limitations, we believe that our findings provide a valuable foundation for future research. We recommend that subsequent studies be designed to further investigate the impact of thrombocytosis and thrombocytopenia in patients with rheumatoid arthritis. Such research could focus on prospective data collection and larger, multicenter cohorts to enhance the reliability of the findings. By understanding the implications of these hematological conditions, timely diagnosis and prompt treatment strategies can be developed, ultimately leading to a reduction in morbidity and mortality among affected patients.

The present study also has several notable strengths that contribute to its value. Firstly, it provides important insights into the prevalence and clinical associations of thrombocytosis and thrombocytopenia in treatment-naïve RA patients, an area that has not been extensively explored in the literature. By focusing on treatment-naïve individuals, the study eliminates potential confounding factors related to previous treatments, allowing for a clearer understanding of the natural disease state. Additionally, the use of established diagnostic criteria for RA, along with thorough clinical assessments, enhances the reliability of the findings. The significant associations identified with age, RA factor positivity, and anti-CCP antibody positivity underscore the relevance of these markers in clinical practice. Furthermore, the study's retrospective design allows for the examination of real-world data, providing a snapshot of current clinical presentations. This can inform clinicians about potential hematological complications in RA patients. Overall, despite its limitations, the study lays the groundwork for future research and emphasizes the need for awareness of thrombocytosis and thrombocytopenia in managing RA.

While this study identifies significant associations between various factors and the outcomes, it is important to clarify that causality cannot be established due to its retrospective design. Emphasizing this limitation strengthens the interpretation of the findings, as the observed relationships may be influenced by confounding variables or other factors. Acknowledging this limitation highlights the need for further research, particularly prospective studies, to explore and establish causal relationships more definitively. Moreover, to enhance the generalizability of the findings, we recommend conducting multicenter studies. By involving diverse patient populations from multiple institutions, future research can capture a wider range of clinical presentations and variations in practice. This approach would validate the results across different settings, addressing potential biases associated with single-institution studies and ultimately increasing the applicability of the findings to broader populations. By designing studies that collect data moving forward, researchers can ensure more accurate and consistent measurements of variables, as well as reduce reliance on existing records, which may be incomplete or subject to recall bias. Furthermore, prospective studies also allow for better control over data collection methods and the timing of assessments, leading to more reliable findings and a clearer understanding of causal relationships in the study population.

## Conclusions

In our study, thrombocytosis was observed in approximately one-third (52, 31.5%) of treatment-naïve RA patients, showing a significant association with age, RAF positivity, and anti-CCP antibody negativity, but not with gender or disease severity. Conversely, thrombocytopenia was relatively uncommon, occurring in only 11 (6.7%) of treatment-naïve RA patients, and it demonstrated no significant associations with age, gender, RAF positivity, anti-CCP antibody positivity, or disease severity. These findings highlight the specific factors associated with thrombocytosis in RA while indicating that thrombocytopenia is less prevalent and not significantly linked to the clinical variables examined. This study provides valuable insights into the clinical characteristics and associations in treatment-naïve RA patients. However, the retrospective design limits the ability to establish causality, and the single-institution nature of the study may affect the generalizability of the results. Future research should consider multicenter approaches and prospective data collection to minimize biases and strengthen the evidence base. Overall, while the study contributes to the existing literature, further investigations are needed to confirm these associations and explore causal relationships in broader, more diverse populations.

## Appendices

Appendix A

The 2010 American College of Rheumatology Criteria for Classifying Rheumatoid Arthritis

The 2010 American College of Rheumatology (ACR) criteria is used for classifying rheumatoid arthritis [14]. The components are as follows:

A. Joint Involvement:

1 large joint (e.g., shoulder, hip) = 0 points

2-10 large joints = 1 point

1-3 small joints (e.g., fingers, wrists) = 2 points

4-10 small joints = 3 points

10 joints (at least one small joint) = 5 points

B. Serology:

Negative rheumatoid arthritis factor (RA Factor) and anti-citrullinated protein (anti-CCP) antibody = 0 points

Low positive RA Factor or Anti-CCP Antibody = 2 points

High positive RA Factor or Anti-CCP Antibody = 3 points

C. Acute Phase Reactants:

Normal C-reactive protein (CRP) and erythrocyte sedimentation rate (ESR) = 0 points

Abnormal CRP or ESR = 1 point

D. Duration of Symptoms:

<6 weeks = 0 points

≥6 weeks = 1 point

*Interpretation: *To classify a patient as having RA, they must score a total of 6 or more points from these criteria.

Appendix B

Disease Activity Score 28 (DAS-28)

The DAS-28 score is a measure used to assess the activity of rheumatoid arthritis and takes into account 28 specific joints and combines various clinical parameters [15]. The components are listed below.

A. Number of swollen joints (out of 28)

B. Number of tender joints (out of 28)

C. Patient's global assessment of health (on a scale from 0 to 100 mm, typically using a visual analog scale)

D. Erythrocyte sedimentation rate (ESR) or C-reactive protein (CRP) level.

The scoring is listed below.

Remission: ≤2.6

Low disease activity: DAS-28 score 2.7-3.2

Moderate disease activity: DAS-28 score 3.3-5.1

High disease activity: DAS-28 score >5.1
